# Modulation of Innate Immune-Related Genes and Glucocorticoid Synthesis in Gnotobiotic Full-Sibling European Sea Bass (*Dicentrarchus labrax*) Larvae Challenged With *Vibrio anguillarum*

**DOI:** 10.3389/fimmu.2018.00914

**Published:** 2018-05-08

**Authors:** Felipe E. Reyes-López, Johan Aerts, Eva Vallejos-Vidal, Bart Ampe, Kristof Dierckens, Lluis Tort, Peter Bossier

**Affiliations:** ^1^Department of Cell Biology, Physiology and Immunology, Universitat Autònoma de Barcelona, Bellaterra, Spain; ^2^Stress Physiology Research Group, Faculty of Pharmaceutical Sciences, Ghent University, Ostend, Belgium; ^3^Stress Physiology Research Group, Animal Sciences Unit, Flanders Research Institute for Agriculture, Fisheries and Food, Ostend, Belgium; ^4^Biostatistics and Data Modeling, Animal Sciences Unit, Flanders Research Institute for Agriculture, Fisheries and Food, Melle, Belgium; ^5^Laboratory of Aquaculture & Artemia Reference Center (ARC), Ghent University, Gent, Belgium

**Keywords:** cytokines, iron regulation, cortisol, gnotobiotic system, European sea bass, fish larvae, *Vibrio anguillarum*

## Abstract

Although several efforts have been made to describe the immunoendocrine interaction in fish, there are no studies to date focusing on the characterization of the immune response and glucocorticoid synthesis using the host–pathogen interaction on larval stage as an early developmental stage model of study. Therefore, the aim of this study was to evaluate the glucocorticoid synthesis and the modulation of stress- and innate immune-related genes in European sea bass (*Dicentrarchus labrax*) larvae challenged with *Vibrio anguillarum*. For this purpose, we challenged by bath full-sibling gnotobiotic sea bass larvae with 10^7^ CFU mL^−1^ of *V. anguillarum* strain HI 610 on day 5 post-hatching (dph). The mortality was monitored up to the end of the experiment [120 hours post-challenge (hpc)]. While no variations were registered in non-challenged larvae maintained under gnotobiotic conditions (93.20% survival at 120 hpc), in the challenged group a constant and sustained mortality was observed from 36 hpc onward, dropping to 18.31% survival at 120 hpc. Glucocorticoid quantification and expression analysis of stress- and innate immunity-related genes were carried out in single larvae. The increase of cortisol, cortisone and 20β-dihydrocortisone was observed at 120 hpc, although did not influence upon the modulation of stress-related genes (*glucocorticoid receptor 1 [gr1], gr2*, and *heat shock protein 70 [hsp70]*). On the other hand, the expression of *lysozyme, transferrin*, and *il-10* differentially increased at 120 hpc together with a marked upregulation of the pro-inflammatory cytokines (*il-1β* and *il-8*) and *hepcidin*, suggesting a late activation of defense mechanisms against *V. anguillarum*. Importantly, this response coincided with the lowest survival observed in challenged groups. Therefore, the increase in markers associated with glucocorticoid synthesis together with the upregulation of genes associated with the anti-inflammatory response suggests that in larvae infected with *V. anguillarum* a pro-inflammatory response at systemic level takes place, which then leads to the participation of other physiological mechanisms at systemic level to counteract the effect and the consequences of such response. However, this late systemic response could be related to the previous high mortality observed in sea bass larvae challenged with *V. anguillarum*.

## Introduction

The innate immune response plays a pivotal role in the activation of the host defense mechanisms and determines the nature of the adaptive immune response (1). The components of the innate immune system are divided into physical (mucus layer, which acts as a physical and chemical barrier) (2); cellular (phagocytic cells such as neutrophils and monocytes/macrophages); and humoral factors (based on pattern-recognition specificities or effector functions) (3). In this framework, the presence of receptors able to activate pathways responsible for the cell signaling cascade are crucial to promote a pro-inflammatory reaction, modulating the innate and adaptive immune response (4). This function is developed by pattern-recognition receptors (PRRs), among others by soluble PRRs such as pentraxins (5). Pentraxins (C-reactive protein and serum amyloid protein) are a phylogenetically conserved superfamily of proteins characterized by the presence of around 200 amino acid–pentraxin domain in their carboxy-terminal region. These proteins are considered lectins acting as a non-redundant component of the humoral arm of innate immunity mediating agglutination, complement activation, and opsonization (6).

Previous reports have shown the expression of antibacterial-related components at the time of hatching and the following weeks (7, 8), indicating that these molecules play a relevant regulatory and effector function to prepare the host against potential environmental pathogens that may be encountered during the early larval development stage when fish immunity is not fully mature. Lysozyme is a bacteriolytic enzyme that hydrolyzes the β-[1,4]-glycosidic linkage of bacterial cell wall peptidoglycans. Its expression on sea bass (*Dicentrarchus labrax*) larvae has been detected at 24 hours post-hatching (9). Another protein involved in the innate humoral response is transferrin. This iron-binding blood plasma glycoprotein controls the level of free iron in biological fluids. Transferrin has a bacteriostatic activity, a property assigned to its iron-binding function (10), thus chelating the available iron necessary for bacterial growth. Transferrin is also considered an acute phase protein (APP) which acts in the inflammatory response to remove iron from the bloodstream (11). Hepcidin, a liver-produced hormone that constitutes the main circulating regulator of iron absorption and distribution across tissues (plasma and intestine) and cells (macrophages, erythrocytes, and hepatocytes) has also been associated with antifungal and antibacterial activity through binding to cell walls (12). Taken together, these proteins share the role of being responsible for iron homeostasis and also participating in the immunity against pathogens.

A key mechanism in the initiation of the antibacterial response is mediated by the expression of pro-inflammatory cytokines involved in the upregulation of inflammatory reactions produced predominantly by activated macrophages. Among them, interleukin-1β (IL-1β) is an endogenous pyrogen produced and released at the early stage response following infections, and subsequently considered as initiator of the pro-inflammatory response in macrophages, activator of lymphocytes and also a synthesis promoter of other cytokines and prostaglandins (13, 14). Interleukin-8 (*IL-8*) is a chemokine produced by macrophages and somatic cells whose primary function is to serve as chemoattractant for neutrophils and T cells to the site of infection. It is also involved in phagocytosis and respiratory burst. Another crucial pro-inflammatory chemokine is chemokine (C–C motif) ligand 4 (CCL4), better known as macrophage inflammatory protein-1β, involved in infection. In mammals, it is produced mainly by macrophages, dendritic cells, and lymphocytes (15).

The pro-inflammatory response is strictly controlled by anti-inflammatory cytokines which regulate the cytokine expression, immune cell proliferation and promote tissue repair (16, 17). The role of interleukin-10 (*IL-10*) has been widely described as a potent anti-inflammatory cytokine that regulates the expression of pro-inflammatory cytokines, contributing to the pathogen infection resolution and also reducing tissue damage caused by inflammation (18, 19). Thus, a highly regulated balance between pro- and anti-inflammatory cytokines makes a successful immune response against pathogens.

Vertebrates under stressful stimuli launch an endocrine stress response. This response comprises an immediate adrenaline res-ponse which prepares the organism for the “fight or flight” reaction by increasing plasma glucose levels and activating cardiovascular responses. In addition, glucocorticoids, in particular cortisol or corticosterone depending on the species, are released through activation of the hypothalamic–pituitary–interrenal (HPI) axis in fish or hypothalamic–pituitary–adrenal axis in other vertebrates (20–22). These plasma glucocorticoids are generally accepted as biomarkers for stress. They mediate a redistribution of energy with the ultimate goal to restore pre-stress conditions and homeostasis, acting as adaptive hormones. Failure to regain homeostasis will lead to chronic stress and maladaptation and eventually to higher susceptibility to pathogens and disease. Thus, when an organism cannot fully recover an allostatic overload is imposed thus becoming prone to detrimental effects of glucocorticoid mediated actions (e.g., immune suppression, decreased growth, impaired reproduction, increased mortality), leading to “distress” and difficulty to regain the set-points for eustress and homeostasis (23, 24). The main corticosteroid hormone in teleosts is cortisol (25) which has become the most common physiological indicator of stress in fish (22). The receptors for cortisol are the glucocorticoid receptor (GR) and mineralocorticoid receptor (MR) (26). In several fish species, including sea bass (27), two GR isoforms (GR1 and GR2) have been reported (28, 29). Importantly, the two GR isoforms differ in glucocorticoid sensitivity and affinity, being GR2 more sensitive to lower concentrations of cortisol (28, 30).

Cortisol is secreted by head kidney interrenal cells from a concatenated response involving the corticotrophin-releasing hormone (CRH) and the adrenocorticotropic hormone (ACTH), produced and secreted in the brain hypothalamic cells and anterior pituitary, respectively. Cortisol is synthesized from cholesterol by its conversion to pregnenolone catalyzed by P450 side chain cleavage, the rate-limiting enzyme in steroidogenesis. Pregnenolone is the precursor of progesterone synthesis that, catalyzed by 17α-hydroxylase, mediates the conversion to 17α-hydroxyprogesterone (17α-HP). The enzyme 21-hydroxylase catalyzes its conversion to 11-deoxycortisol, and 11β-hydroxylase is the enzyme responsible for the terminal step catalyzing the conversion from 11-deoxycortisol to cortisol (31–33). The main cortisol phase I metabolites are cortisone and 20β-dihydrocortisone. In fact, large circulating concentrations of cortisone have been detected in fish subjected to stress (34). It has been proposed that the conversion from cortisol to cortisone, mediated by 11β-hydroxysteriod dehydrogenase type 2, serves to downregulate cortisol into a non-active keto-metabolite. However, this hypothesis has been less studied to date (24).

It has been extensively described that stress and immune response are tightly connected (35, 36). Many studies have reported that a stressor induces alterations on innate immune response (37–39). Thus, it is widely accepted that cortisol inhibits the release of pro-inflammatory cytokines (40–42), probably as a mechanism for control and resolution of inflammation. Nowadays, as far as we know, there are no studies focused on host–pathogen interaction in which an association between glucocorticoid synthesis and immune response has been evaluated in larvae after a challenge with a highly pathogenic bacterium. In this study, the use of a gnotobiotic culture system allows to evaluate the specific and direct interaction between the microorganism of interest and the host, thus avoiding the intrinsic microbial community dynamics (43, 44). Therefore, the aim of this study was to evaluate the glucocorticoid profile and the modulation of stress- and innate immune-related genes in gnotobiotic full siblings in specific time points related to mortality of European sea bass larvae challenged with *Vibrio anguillarum*. The kinetics of key actors associated with the innate immune response will be useful to understand the ability of fish larvae to activate immune protective-related mechanisms against *V. anguillarum* and the endocrine-mediated response by glucocorticoid synthesis as an orchestrated systemic response at early larvae stage and under controlled microbial experimental conditions.

## Materials and Methods

### Bacterial Strains and Culture Conditions

The rifampicin-resistant *V. anguillarum* strain HI 610 (O. Bergh, Institute of Marine Research, Norway), isolated by natural selection, was used in this study. The rifampicin resistance allows this strain to survive in an axenic sea bass larvae culture water environment. The bacteria were grown overnight at 28°C on 10% marine broth (Difco Laboratories) with NaCl to obtain the same salinity as the water in the fish larvae experiment (36 g L^−1^) and supplemented with 10 ppm rifampicin (Sigma). The bacterial suspension was then centrifuged at 1,500 × *g* for 10 min at 4°C and resuspended in distilled water added with Instant Ocean artificial sea salt (Aquarium Systems) to obtain a salinity of 36 g L^−1^, and supplemented with 10 ppm rifampicin and kept on a horizontal shaker at 150 rpm at 16°C. The bacterial suspension density was determined spectrophotometrically (Genesys 20, Thermospectronic) at 550 nm according to the McFarland standard (BioMérieux).

### Challenge Tests With *V. anguillarum* and Gnotobiotic Full-Sibling Sea Bass Larvae

**Figure 1:**
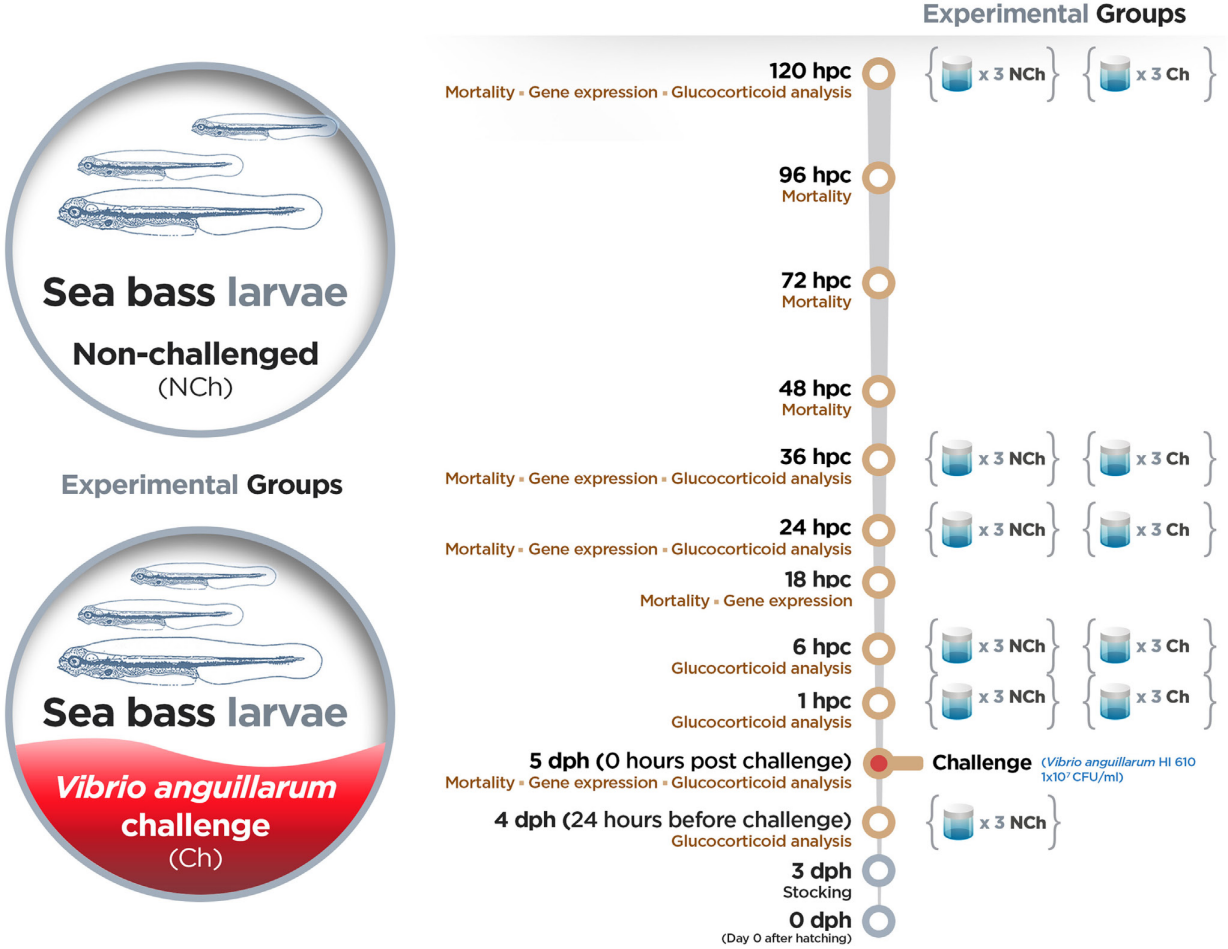
Experimental setup.

Upon arrival, sea bass eggs were acclimatized in UV-sterilized seawater for 4 h in a cylindro-conical tank. The water temperature (16 ± 1°C) and salinity (36 g L^−1^) were kept constant during the experiment. The disinfection of eggs, hatching and axenity tests were performed according to Dierckens et al. (43). All larvae analyzed in this study belonged to the same full-sibling family batch. A summary of the experimental setup with the assays and time points evaluated is given in Figure 1. On day 3 dph, full-sibling larvae were stocked in groups of 12 larvae in 10 mL sterile screw cap vials with the addition of 10 mg L^−1^ rifampicin. Three vials (replicates) were prepared for each treatment and time point included in the study. Full-sibling larvae were challenged by bath with 10^7^ CFU mL^−1^ of *V. anguillarum* strain HI 610 on 5 dph in a gnotobiotic system. The mortality was monitored in all vials by counting the living larvae (transparent and swimming) under a dissecting microscope at 24 hours before challenge (hbc) and at 0, 18, 24, 36, 48, 72, 96, and 120 hpc. As control group, uninfected larvae (mock-challenged) were used, and whole-body larva sampling (10 larvae per condition) was performed at the same time points mentioned above. Larvae were not fed during the experiment. Larvae were collected, snap-frozen in liquid nitrogen and kept at −80°C until analysis. Embryos were sacrificed by over-anesthetization using methylsulfonatetricaine (MS-222) (Sigma) and immediately sampled. After sampling, larvae were immediately frozen in liquid nitrogen and stored at −80°C until analysis.

### Ethics Statement

All experiments were approved by the Ethical Committee of the Faculty of Veterinary Medicine and the Faculty of Bioscience Engineering, Ghent University (no. EC2015_02) and carried out in accordance with the recommendations of the European Union Ethical Guidelines for experimental animal care and other scientific purposes (2010/63/EU).

### Glucocorticoid Quantification

As the pertinent literature lacks a method for analyzing a full glucocorticoid profile in a single fish larva (whole body), a recently validated ultra-performance liquid chromatography coupled to tandem mass spectrometry (UPLC–MS/MS) quantification method was followed (44). Shortly, a single sea bass larva was sampled, rinsed with ultrapure water, dried on a paper tissue, and subsequently weighed. The larva was homogenized and HPLC-gradient grade methanol (VWR) was used as extraction solvent. Purification was done using GracePure™ SPE C18-Max 500 mg/6 mL solid-phase extraction (SPE) columns. After resuspension, UPLC–MS/MS was used to quantify the glucocorticoid profile for the active hormone cortisol, its precursors (17α-HP and 11-deoxycortisol), and phase I metabolites (cortisone, 20β-dihydrocortisone, tetrahydrocortisol, and tetrahydrocortisone).

Single whole-body larva samples (*n* = 10 per time point and experimental condition) were taken at 24 hbc from non-treated larvae, and also at 0, 1, 6, 24, 36, and 120 hpc from the non-challenged (mock-infected) and challenged larvae. Glucocorticoid quantification was carried out at 24 hbc to evaluate whether larval manipulation induced stress by handling at 24 h after finishing the stocking process. The glucocorticoid quantification was also carried out at 0, 1, 6, 24, 36, and 120 hpc to evaluate the potential capability of bacteria to induce an acute stress response (0, 1, 6, and 24 hpc) and based on previous studies conducted in European sea bass that showed the cortisol peak at 1 h after exposure to an acute stressor (27) as well as in studies conducted in larvae of other fish species such as rainbow trout (1 h post stress) (45) and red drum (1 h post stress) (46). The analysis of 24, 36 and 120 hpc time points was also used to evaluate the modulation of stress- and immune-related genes.

### RNA Isolation and Complementary DNA (cDNA) Synthesis

Total RNA was isolated from a single whole larva using TRI reagent (Sigma) according to the manufacturer’s instructions with some modifications. RNA was precipitated from the aqueous solution with 2-propanol (Sigma) in presence of 10 µg of glycogen (Sigma) and incubated for 1 h at −80°C. The RNA pellet was dissolved in 10 µL of nuclease free-water and immediately stored at −80°C until use. The RNA concentration was determined using a NanoDropND-2000 spectrophotometer (Thermo Scientific), and the integrity was checked by Experion RNA StdSens analysis (Bio-Rad Laboratories). Samples with an RNA quality indicator number greater than 8.0 were chosen for gene expression analysis. Total RNA (500 ng) was used as template to synthesize cDNA using iScript cDNA kit (Bio-Rad Laboratories) according to the manufacturer’s instructions.

### Gene Expression Analysis

Real-time PCR assay was carried out to analyze the expression pattern of different stress and immune relevant genes in gnotobiotic full-sibling European sea bass larvae challenged with *V. anguillarum*. Samples (*n* = 10) were taken randomly at 0 hpc from non-treated larvae, and samples were taken both from the non-challenged (mock-infected) (*n* = 10) and challenged (*n* = 10) larvae at 18, 24, 36, and 120 hpc. Several reference candidate genes [ribosomal protein l13 (*rpl13*), elongation factor 1α (*ef1*α), and 40S ribosomal protein SA (*rpsa*)] were tested using the BestKeeper software (47). According to previous antecedents on sea bass infected with *V. anguillarum* (48), the *rpl13* was chosen in our study as reference gene because its lower variation tested upon all the samples included in our study. Specific primers used for gene expression analysis (Table 1) were designed with Primer-Blast. The potential primer secondary structures and primer specificity and was checked with OligoAnalyzer (version 3.1) and Primer-Blast, respectively. Real-time PCR reactions were performed with iTaq universal sybr green supermix (Bio-Rad Laboratories) using 2.5 µL of 1:40 dilution of cDNA (for genes of interest) or 2.5 µL of 1:1,000 dilution (for reference gene). Primers for all genes were used at 500 nM. RNA sample mix (prepared from 1 µL of each total RNA sample stock), cDNA synthesis mix, and MilliQ-water (same than used to prepare the mix for all primers evaluated) were used as real-time PCR internal amplification controls. The thermal conditions used were as follows: 3 min at 95°C of pre-incubation followed by 40 cycles at 95°C for 30 s and 60°C for 30 s. An additional temperature ramping step was utilized to produce melting curves from 65 to 95°C to verify amplification of a unique single product on all samples. All the reactions were performed in duplicate using a CFX384 Touch Real-Time PCR Detection System (Bio-Rad Laboratories). The quantification was done according to Pfaffl method corrected for efficiency of each primer set (49). The value for each experimental condition was expressed as normalized relative expression, calculated in relation to values of control group and normalized against those of the reference gene. The results were expressed as average of values obtained at 0, 18, 24, 36, and 120 hpc (*n* = 10 larvae per condition group and time point).

**Table 1:** Primers used in real-time PCR for gene expression analysis.

Gene	Primer sequence (5′–3′)	Accession number	Amplicon size	Efficiency
*pentraxin*	Fw: 5′-AGTTTTTGCTGCTGGTGGTG-3′ Rv: 5′-GCCAAAGAGAAAAGGACGTGG-3′	EU660933.1	199	1.95
*lysozyme*	Fw: 5′-TGATGCAGGTTGTTGATGTTAATC-3′ Rv: 5′-TCCATCCCCCATATTGTAGGC-3′	KJ433681.1	194	1.94
*transferrin*	Fw: 5′-GCCCCCAAACACAGATTCCT-3′ Rv: 5′-CCGTCAGCACCCATACTGTT-3′	FJ197144.1	177	1.95
*hepcidin*	Fw: 5′-GGAATCGTGGAAGATGCCGT-3′ Rv: 5′-CAGACACCACATCCGCTCAT-3′	DQ131605.1	108	1.86
Interleukin (*il*)*-1β*	Fw: 5′-ATCTGGAGGTGGTGGACAAA-3′ Rv: 5′-AGGGTGCTGATGTTCAAACC-3′	AJ269472.1	106	2.02
*il-8*	Fw: 5′-GTCTGAGAAGCCTGGGAGTG-3′ Rv: 5′-GCAATGGGAGTTAGCAGGAA-3′	AM490063.1	110	1.98
Chemokine (C–C motif) ligand 4 (*ccl4*)	Fw: 5′-TCCTCGTCTCACTCTGTCTGT-3′ Rv: 5′-GACCTGCCACTGTCTTCAGC-3′	AM490064.1	197	1.95
*il-10*	Fw: 5′-CGACCAGCTCAAGAGTGATG-3′ Rv: 5′-AGAGGCTGCATGGTTTCTGT-3′	AM268529.1	199	2.06
Glucocorticoid receptor 1 (*gr1*)	Fw: 5′-GAGATTTGGCAAGACCTTGACC-3′ Rv: 5′-ACCACACCAGGCGTACTGA-3′	AY549305	401	1.92
Glucocorticoid receptor 2 (*gr2*)	Fw: 5′-GACGCAGACCTCCACTACATTC-3′ Rv: 5′-GCCGTTCATACTCTCAACCAC-3′	AY619996	403	1.97
Heat shock protein 70 (*hsp70*)	Fw: 5′-GCTCCACTCGTATCCCCAAG-3′ Rv: 5′-ACATCCAGAAGCAGCAGGTC-3′	AY423555.2	172	1.94
Ribosomal protein l13 (*rpl13*)	Fw: 5′-AAGGGAGACAGCACTGAGGA-3′ Rv: 5′-TGCCAAAAAGACGAGCGTTG-3′	DQ836931.1	175	2.00
Elongation factor 1α (*ef1*α)	Fw: 5′-AACTTCAACGCCCAGGTCAT-3′ Rv: 5′-CTTCTTGCCAGAACGACGGT-3′	AJ866727.1	144	1.97
40S ribosomal protein SA (*rpsa*)	Fw: 5′-TGATTGTGACAGACCCTCGTG-3′ Rv: 5′-CACAGAGCAATGGTGGGGAT-3′	HE978789.1	79	1.93

### Statistical Analysis

Cumulative survival was analyzed by a linear regression model with Bonferroni posttest. Gene expression analysis of immune-related genes in whole-body larva and the glucocorticoid screening was analyzed by a linear regression model with treatment, time, and their interaction as fixed effects. The outcomes were log-transformed to obtain normality. Normality of the analyzed outcomes was evaluated based a graphical examination of the residuals. In all analyses, a *p*-value < 0.05 was considered statistically significant. In case of significant effects, a *post hoc* test for treatment (with Tukey correction) was performed (at each time point). The analysis was carried out with SAS 9.4 for Windows.

## Results

### Cumulative Survival in Gnotobiotic Full-Sibling European Sea Bass Larvae Challenged With *V. anguillarum*

**Figure 2:**
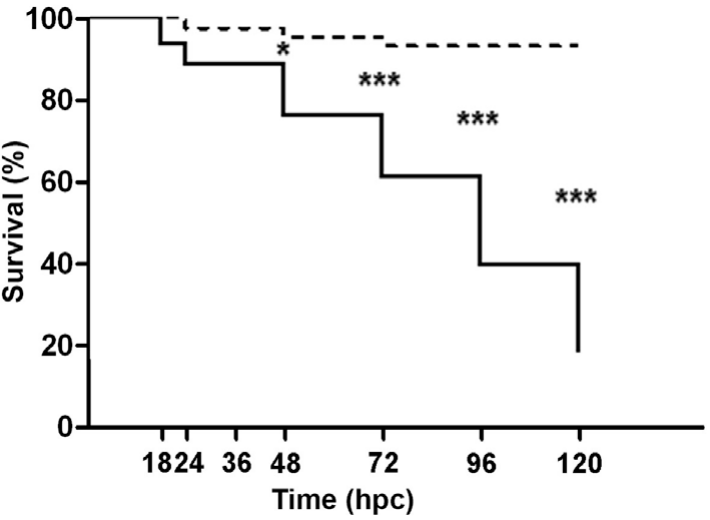
Cumulative survival of full-sibling sea bass larvae challenged with *Vibrio anguillarum*. The mortality (mean ± SE) is represented in each time [hours post-challenge (hpc)] for non-challenged (dashed line) and challenged group (continuous line). Significant differences (linear regression model with Bonferroni posttest) are shown with asterisks (*p < 0.05; ***p < 0.001).

To evaluate whether *V. anguillarum* challenge had an effect on larval viability, the cumulative survival was recorded throughout the study (Figure 2). Almost no variations were registered in non-challenged larvae maintained under gnotobiotic conditions, whose cumulative survival decreased to 93.20% at 120 hpc. By contrast, a gradual decline on larval survival was observed from the moment of exposition to the bacterial pathogen (93.94% survival) to 24 hpc (88.52% survival). This decrease in larval survival ceased between 24 and 36 hpc. However, from 36 hpc onward, a constant and sustained drop in the cumulative survival was observed in larvae challenged with *V. anguillarum* resulting in a significant difference at 48 (76.31% survival), 72 (61.05% survival), 96 (39.68% survival), and 120 hpc (18.31% survival) compared with non-challenged larvae. This indicates that the challenge with *V. anguillarum* provokes a systemic failure of the larval defense systems resulting in death when cultured under gnotobiotic conditions.

### Glucocorticoid Quantification in Gnotobiotic Full-Sibling European Sea Bass Larvae Challenged With *V. anguillarum*

**Figure 3:**
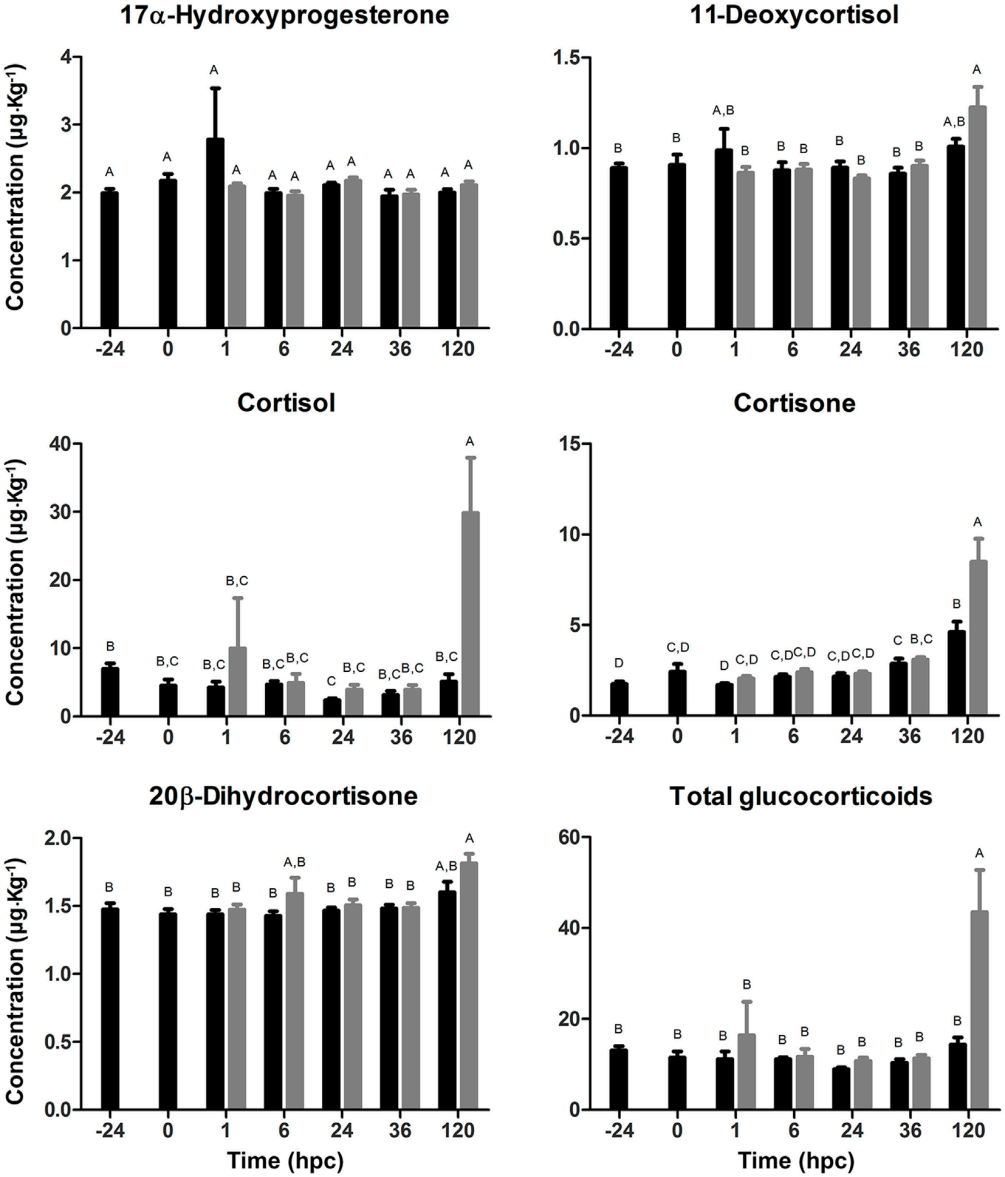
Glucocorticoid profile (mean ± SE) in full-sibling sea bass larvae challenged with *Vibrio anguillarum*. The amount (μg kg^−1^ whole body) of 17α-hydroxyprogesterone, 11-deoxycortisol, cortisol, cortisone, 20β-dihydrocortisone, and total glucocorticoids was quantified from whole-single larva (*n* = 10 per time point) in each time [hours post-challenge (hpc)] for non-challenged (black bar) and challenged group (gray bar). Significant differences (linear regression model with Tukey correction posttest) are shown by different capital letters (A, B; *p* < 0.05).

To evaluate whether the challenge with *V. anguillarum* had a stimulatory secretory effect on either cortisol, its precursors, and the most important phase I metabolites, a glucocorticoid profile was quantified in whole body of a single larva challenged with *V. anguillarum*. In addition, the glucocorticoid profile was evaluated at 24 hbc to determine whether larval manipulation induced stress by handling after finishing the stocking process (Figure 3). No variations were recorded on the synthesis of the glucocorticoid precursors, 17α-HP and 11-deoxycortisol; neither at 24 hbc nor in the non-challenged or challenged groups. Cortisol synthesis showed no significant differences in both experimental groups in the first 36 hpc. However, a marked time-dependent increase was observed in larvae challenged with *V. anguillarum* at 120 hpc (29.849 ± 8.102 μg L^−1^) compared with challenged larvae at 36 hpc (3.919 ± 0.654 μg L^−1^). The cortisol mean value at 120 hpc in challenged larvae was also higher than non-challenged larvae at the same time (5.099 ± 1.077 μg L^−1^), whose cortisol mean value remained unaltered at all time points evaluated. Because of the high level of cortisol at 120 hpc in the challenged group, we also evaluated the synthesis of phase I metabolites to obtain an even more detailed view on cortisol metabolism. In concordance with the cortisol value, cortisone showed also the highest level at 120 hpc in the challenged group (8.478 ± 1.288 μg L^−1^) compared with challenged larvae at 36 hpc (3.089 ± 0.148 μg L^−1^). This cortisone value at 120 hpc in the challenged group was also higher than in the non-challenged group at the same time point (4.621 ± 0.566 μg L^−1^). As in the case of the challenged group, cortisone in the non-challenged group was higher at 120 hpc than 36 hpc (2.871 ± 0.277 μg L^−1^). On the other hand, the synthesis of 20β-dihydrocortisone did not vary in the non-challenged group, observing only an increase at 120 hpc (1.813 ± 0.071 μg L^−1^) compared with 36 hpc (1.485 ± 0.036 μg L^−1^) in the challenged group. Tetrahydrocortisol and tetrahydrocortisone were absent in all samples, indicating that the latter were free of contamination caused by exogenous glucocorticoids from hands, water, etc. The total glucocorticoid concentration, being the sum of all glucocorticoids quantified, at 120 hpc (43.477 ± 9.299 μg L^−1^) increased compared with 36 hpc in the challenged group (11.365 ± 0.783 μg L^−1^), and at 120 hpc in the non-challenged group (14.331 ± 1.614 μg L^−1^). In all, this suggests an increased synthesis of cortisol at 120 hpc in sea bass larvae challenged with *V. anguillarum* whereby the glucocorticoid level is most likely correlated with the circulating concentrations of cortisone, affecting in sum to the total glucocorticoid circulating concentrations in challenged sea bass larvae.

### Gene Expression in Gnotobiotic Full-Sibling European Sea Bass Larvae Challenged With *V. anguillarum*

**Figure 4:**
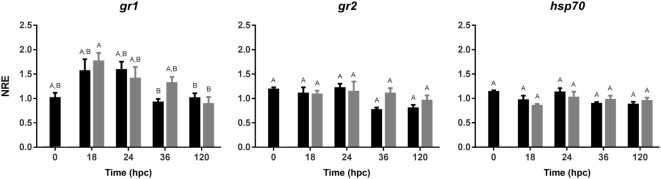
Normalized relative expression (NRE) analysis (mean ± SE) of stress-related genes in full-sibling sea bass larvae challenged with *Vibrio anguillarum*. The expression of glucocorticoid receptor 1 (*gr1*), *gr2*, and *heat shock protein 70 (hsp70)* was evaluated from whole-single larva (*n* = 10 per time point) in each time [hours post-challenge (hpc)] for non-challenged (black bar) and challenged group (gray bar). Significant differences (linear regression model with Tukey correction posttest) are shown by different capital letters (A, B; *p* < 0.05).

The expression profile was analyzed to evaluate the time-dependent modulation of stress- and innate immune-related genes in larvae challenged and non-challenged with *V. anguillarum*. The expression of stress-related genes was evaluated in sea bass challenged with *V. anguillarum* (Figure 4). The glucocorticoid receptor *gr1* did not vary its expression when the mRNA abundance between the non-challenged and challenged groups was compared in all time points tested. Importantly, no modulations were either registered for *gr2*. The expression of *hsp70* was not either modulated on sea bass larvae when exposed to *V. anguillarum*, thus remaining the expression at basal level in all time points evaluated. These results indicate that at 120 hpc cortisol did not exert any modulatory effect on the expression of gr1, gr2, and hsp70 in sea bass larvae challenged with *V. anguillarum* at those time points.

**Figure 5:**
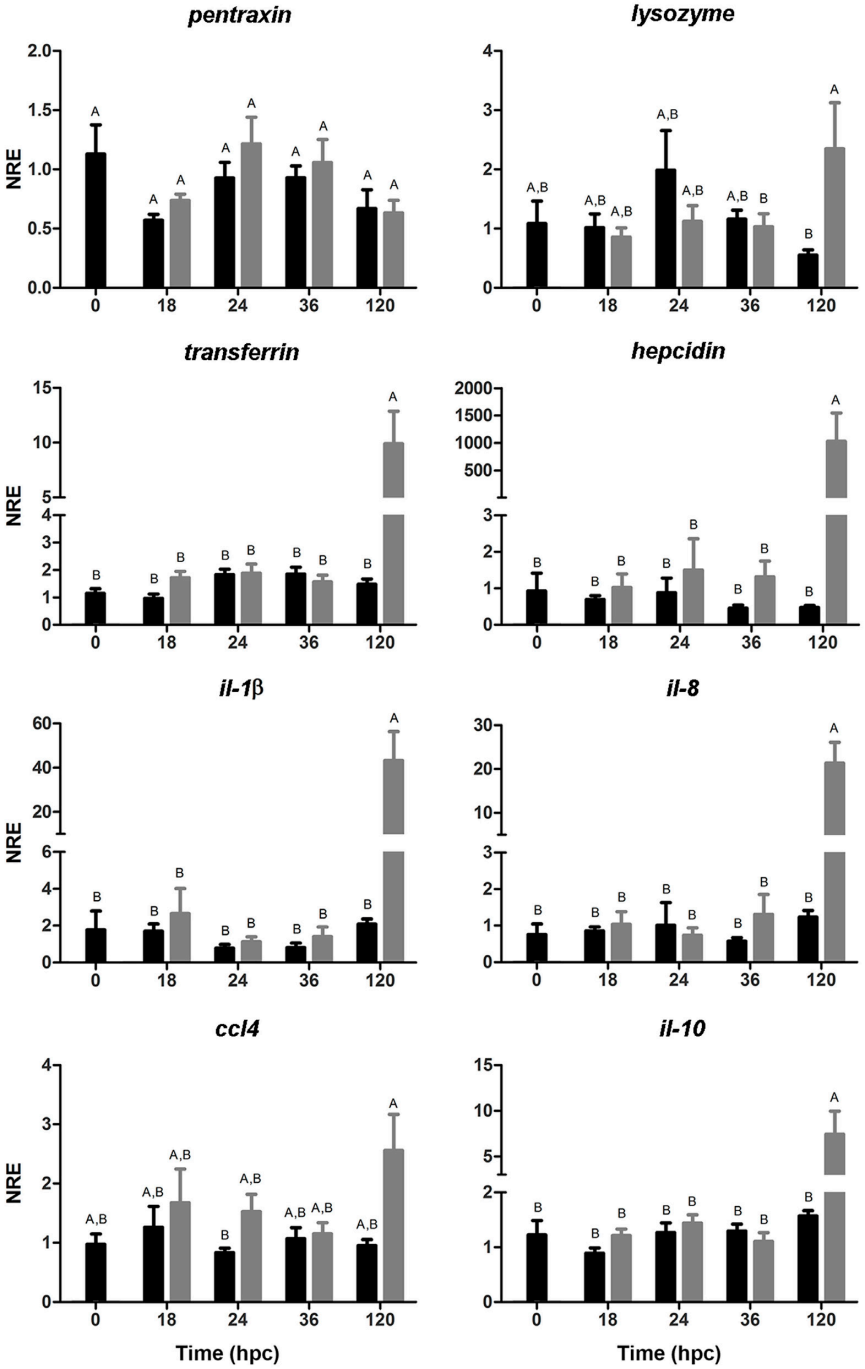
Normalized relative expression (NRE) analysis (mean ± SE) of immune-related genes in full-sibling sea bass larvae challenged with *Vibrio anguillarum*. The expression of *pentraxin*, lysozyme, *transferrin, hepcidin, Il-1β*, il*-8, il-10*, and *ccl4* was evaluated from whole-single larva (*n* = 10 per time point) in each time [hours post-challenge (hpc)] for non-challenged (black bar) and challenged group (gray bar). Significant differences (linear regression model with Tukey correction posttest) are shown by different capital letters (A, B; *p* < 0.05).

The expression of genes associated with the humoral arm of the innate immunity was evaluated (Figure 5). *pentraxin* showed no significant differences between challenged and non-challenged larvae along the time points evaluated in this study. The expression of lysozyme showed no variations during the first 36 hpc; by contrast, the challenged group showed upregulation of lysozyme at 120 hpc compared with the non-challenged group. The expression of *transferrin* remained unaltered up to 36 hpc but increased at 120 hpc compared with the control group. Importantly, the expression of *hepcidin* was markedly augmented (1,027-fold increase) in the group challenged with *V. anguillarum*. In all, these results suggest the increase in the expression of genes associated with bacterial clearance at 120 hpc in sea bass larvae challenged with *V. anguillarum*.

To evaluate whether the upregulation of genes associated with antibacterial activity was correlated with the expression pro-inflammatory genes, the expression of *il-1β* and *il-8* was analyzed. According to the innate immune-related genes, the same expression pattern was observed both in *il-1β* and *il-8* observing a marked increase at 120 hpc in the challenged group. The same trend, although not significant, was observed at 120 hpc in *ccl4*. On the other hand, the expression of *il-10*, an anti-inflammatory cytokine involved in the pro-inflammatory outcome control, showed no variations during the first 36 hpc but its expression increased at 12 hpc in larvae challenged with *V. anguillarum*. Hence, these results suggest that both the pro-inflammatory response and its regulatory mechanism took place at 120 hpc in sea bass larvae challenged with *V. anguillarum*.

## Discussion

In this study, we evaluated the modulation of innate immune-related genes and glucocorticoid synthesis in gnotobiotic full-sibling European sea bass larvae challenged with *V. anguillarum*. Importantly, significant differences in the expression of innate immune-related genes and glucocorticoid profile in gnotobiotic full-sibling European larvae were only observed at 120 hpc, coinciding with the highest difference in the cumulative survival between challenged and non-challenged larvae. Taking together, these results suggest an immune-endocrine association in response to the challenge with *V. anguillarum*. This is the first report in which such response is shown during the first developmental fish stages.

The gnotobiotic system involves absence of cultivable bacteria, thus allowing the specific study of the host–microbial interaction. This larval rearing system avoids the intrinsic dynamics of the conventional environment in terms of microbial community interactions (43). Subsequently, the use of gnotobiotic conditions provides information about the specific and direct interaction between an external bacterial stimulus and the host (50). Thus, the gnotobiotic culture system is an important alternative to solve the problems of a reproducible experimental setup by generating high inter-individual and inter-batch variability in the composition of the standing microbial community (51).

Infectious diseases are a huge problem in aquaculture, causing important economic losses due to high mortality. Vibriosis, an infectious disease whose etiological agent is *V. anguillarum*, has been considered one of the most important mariculture fish diseases (52). Several studies have reported the susceptibility of sea bass to *V. anguillarum* (53, 54). In our results, no significant variations were observed in the cumulative survival in non-challenged groups along the study. By contrast, the challenge with *V. anguillarum* provoked a gradual decline of the sea bass larvae cumulative survival in the first hours post-challenge that then seemed stabilized at 36 hpc. However, a marked and strongest decrease in cumulative survival was also observed from 36 hpc up to the end of the experiment. The low survival of challenged larvae obtained in our study compared with higher survival observed in previous reports in sea bass larvae challenged with *V. anguillarum* strain HI 610 under gnotobiotic conditions (43) could be associated with the more limited larval genetic variability. It may be considered that all the individuals are full-sibling in our study, and hence, a higher effect is recorded as the full-sibling fish are more susceptible to *V. anguillarum*. In teleosts, it has been demonstrated that the same pathogen can differentially affect the cumulative survival on several full-sibling groups, and these differences may reside in their particular ability to mount an efficient immunological response against the pathogen (55).

The expression of genes related to the adaptive immune response has been reported from few days post-hatching onward (8, 50, 56, 57). At cellular level, it is accepted that T cells are the first lymphoid-cell type to appear during fish ontogenesis. The presence of thymocytes in sea bass was detected by immunocytochemistry primarily from 30 dph in thymus (58) and gut (59), while earlier T cells detection by flow cytometry were described from 5 to 12 dph onward (60). However, during this developmental life stage, thymus is not yet a differentiated lymphoid organ (58, 61), suggesting a very early/pre-T cells that could represent a cell type different from the large granular lymphocytes (62). On the other hand, mature B-cells and immunoglobulin M (IgM) were detected in sea bass from 50 dph onward (61, 62), although the presence of maternal IgM has been observed in the sea bass eggs and embryos (61, 63). Thus, the presence of an adaptive (and therefore a mature) immune system can take place from 50 dph onward. These antecedents would indicate that fish depend mainly on innate defense, at least during the first days post-hatching (64–68). Moreover, the presence of innate immune components such as cathepsin, lectins, and lysozyme have been detected at very early stages in oocytes, fertilized eggs, and larval stages of several fish species including sea bass (9, 69, 70).

The innate immune system is of key importance in combating infections by microorganisms in lower vertebrates, particularly under poikilothermic conditions (3). Thus, the modulation of genes associated with innate immunity seems to be of primary importance in the sea bass larvae response against *V. anguillarum*. In our study, the evaluation of genes associated with the stress and the innate immune response was based on the analysis of the most critical survival time points on sea bass larvae challenged with *V. anguillarum* (1): the beginning of a reduction on the cumulative survival and (2) the lowest cumulative survival time point. It has previously been reported that the evaluation of the first time points of infection is critical for fish immunity and, in fact, may even allow to classify phenotypes of responses based on the immune gene expression patterns (55). In the context of innate immunity, the expression of *pentraxin* was evaluated. Pentraxins are lectins present in the body fluids and are associated with the acute phase response (APR) by its role in mediating agglutination, complement activation, and opsonization (6, 71, 72). Our results showed no variation on *pentraxin* gene expression. In teleosts, the upregulation of *pentraxin* has been reported in juvenile ayu following *V. anguillarum* intraperitoneal challenge (73). However, it has been proposed that the level of pentraxin may or may not be elevated during an APR (3). Thus, *pentraxin* expression in the sea bass larvae evaluated in our study seems not to be affected by *V. anguillarum*, suggesting that other PRRs and actors of the innate immunity humoral arm could be implicated in the recognition, and therefore in the response against *V. anguillarum*.

Other proteins involved in the innate humoral innate response were also evaluated. Lysozyme is an enzyme able to hydrolyze the β-[1,4]-glycosidic bond present on peptidoglycans bacterial cell wall. The expression of lysozyme has been detected at 24 hph (9) probably as a primary defense mechanism previous to the first exogenous feeding. In our results, the expression of lysozyme was augmented at 120 hpc, indicating that probably an early recognition of *V. anguillarum* by the host is not taking place. In the same direction, the gene expression of molecules involved in the iron homeostasis regulation such as *transferrin* (with bacteriostatic activity) (10) and *hepcidin* (with antimicrobial activity) (12) were also upregulated at 120 hpc. The fight for iron availability in the bloodstream is crucial to establish the ability of the host to overcome a pathogen threat. Iron is critical for all bacteria to grow and determines the infective success. Thus, the host iron regulation is a key step directly associated with defense mechanisms mounted in response to a bacterial challenge. In our results, the upregulation of *transferrin* and *hepcidin* at 120 hpc could be directly related with one of the strategies responsible for counteracting the mortality observed at 120 hpc. Previous studies have shown that synthetic *hepcidin* induced protection of sea bass challenged against *V. anguillarum* (74). This antecedent suggests that the delayed upregulation of innate-related immune genes involved in the response against *V. anguillarum* could be one of the possible causes of the high mortality observed in our study. This response may also generate a delay in mobilizing the necessary physiological mechanisms to sequester the available iron from the bloodstream. Accordingly, in marine fish, it has been reported that *V. anguillarum* mediates the iron-uptake system by plasmid-mediated pJM1 (75) or from ferric citrate (76). This mechanism allows the bacteria to establish the infection and even can cause fish death as a consequence of septicemia (77). In the last years, increasing attention has been directed to exploit the bacterial weaknesses by modifying iron bioavailability to take advantage of the host iron-uptake system as a control method for bacterial infections (50). In our study, the delayed modulation of *transferrin* and *hepcidin* suggests that the iron-uptake regulation system is crucial for a successful infective process resulting in high mortalities in sea bass larvae.

The modulation of these host iron regulator genes is directly associated not only to their role as innate immune mediators responsible for removing iron from the bloodstream but also with their role as bacteriostatic and antimicrobial agents, acting as positive APPs (78). The APR is a prominent systemic reaction to a particular set of stimuli that may cause disturbance of the homeostasis, for instance, an infection process, resulting in the production of pro-inflammatory cytokines. In fact, Atlantic cod (*Gadus morhua*) injected with heat-killed *V. (Listonella) anguillarum* showed upregulation of *transferrin* accompanied by the expression of APR genes together with upregulation of *il-1β* and *il-8* (79). Accordingly, in our results the upregulation of humoral innate effectors (*lysozyme, transferrin*, and *hepcidin*) is directly related to the upregulation of both *il-1β* and *il-8* at 120 hpc. Regarding pro-inflammatory cytokines, the expression of *il-1β* after intraperitoneal (i.p.) infection with *V. anguillarum* has been previously observed at systemic level in gilthead sea bream (*Sparus aurata*) 4 h after bacterial challenge (80). An upregulation of *il-1β* has also been reported in juvenile sea bass intraperitoneally injected with *V. anguillarum* both at local (skin) and systemic level (spleen, head kidney) mainly during the first 8 h post-infection (81). The upregulation of *il-1β* was also accompanied by the expression of *il-8* but also *il-10* at local (gills) and systemic (head kidney, spleen) sites 24 h after i.p. injection with formalin-killed *V. anguillarum* (82). The joint upregulation of pro-inflammatory (*il-1β, il-8*) and anti-inflammatory (*il-10*) cytokines were also noted in our study at 120 hpc. The pro-inflammatory (involved in the upregulation of inflammatory reactions) and anti-inflammatory (control the pro-inflammatory cytokine response by immunoregulatory molecules) cytokines are relevant actors in the host immunity. In mammals, it has been widely described that IL-10 acts as a potent anti-inflammatory cytokine that regulates and inhibits the expression of pro-inflammatory cytokines, contributing to the normal resolution of infection and reducing tissue damage caused by inflammation (18, 19). In teleost fish, several reports indicated a role of IL-10 as anti-inflammatory cytokine and the induction of high levels of *il-10* in infected fish (55, 83, 84). A sequence analysis performed in cod has shown that *il-1β, il-8*, and *il-10* promoter regions contain similar regulatory domains which can explain the modulation of these genes, similarly of what has been described in mammals (82). This information opens the possibility that the same phenomena occur in sea bass and, therefore, it can be the responsible mechanism for the *il-1β, il-8*, and *il-10* modulation observed at 120 hpc. On the other hand, in mammals, it has been reported that hepcidin can also act as an anti-inflammatory agent inducing a signal cascade by hepcidin-activated Jak2, which phosphorylates the transcription factor Stat3, subsequently provoking an anti-inflammatory transcriptional response by negative feedback (85). Altogether, it seems clear that *V. anguillarum* modulates the expression of pro- and anti-inflammatory cytokines. However, it is striking that a significant regulation of innate immune-related genes takes place at 120 hpc considering the high mortality observed at the same time point. In this aspect, the non-significant differences observed during the first 36 hpc at gene expression level, can also be related to the fact that no significant differences were also observed in the cumulative survival at the same time-period. On the other hand, the non-modulation of genes associated with the immune system in the first 36 hpc could be related to the participation of maternally transferred immune factors devoted to protect early fish stages against invading pathogens before full maturation of immunological systems. Importantly, it has been previously reported that *V. anguillarum* evades the immune response of sea bass (80 g mean weight) (86). Hence, the sum of an immature immune system, the late immune response at 120 hpc together with *V. anguillarum* escape ability from the host antimicrobial defense, could explain the high mortality observed in our study. More studies are needed to elucidate whether the modulation of genes related to innate immune response begin from 48 hpc when differences in the cumulative mortality between challenged and non-challenged groups are observed.

An external stimulus present in the environment, such as a bacterial pathogen, may be sensed by the fish and eventually trigger an immune response, as has been observed in our study. In addition, it can also induce a subsequent global neuroendocrine response when the alarm messengers will reach and activate the HPI axis. This neuroendocrine signaling will induce a stress response in the organism. In teleosts, cortisol is the main glucocorticoid and the final product of the HPI axis activation in response to stressor stimuli exposure (87). Taking into account that cortisol alone provides a good but incomplete snap-shot of HPI axis activity when analyzing whole body of fish larvae, the additional analysis of its direct precursors and phase I metabolites as analyzed in our study provide a more detailed and accurate view of HPI axis effector activity. As described by Øverli et al. (88), reactions to stress vary between individuals as reflected in a proactive or a reactive coping style. As glucocorticoid quantification was done using a single larva, the glucocorticoid production elicited by a different coping style of the individual larvae could be quantified.

Previously, the role of cortisol and its effects in the immune response has been extensively analyzed (36). The previous hypothesis on the variations of immune and endocrine molecules assumes that organisms have a complex physiological machinery to respond efficiently to external stimuli. Hence, it should be taken into account that during larval stages, the full physiological and immune equipment to cope with external challenges may not be complete or they become progressively functional as time-course progresses. Thus, most data available related to hormonal responsiveness of larvae studied so far show that fish larvae are able to secrete cortisol from very early stages, being either from maternal sources in the egg at first hours post-hatching or afterward by the own larval machinery. Thus, Stouthart et al. (89) showed increase of ACTH and cortisol in carp (*Cyprinus carpio*) 24 h post-hatching and Laiz-Carrión et al. (90) showed ACTH activity already at hatching stage. In sea bass, cortisol secretion is observed at first feeding (11 days post-hatching) and a high response at flexion (25 days post-hatching) (27). The marked increase of cortisol levels in sea bass larvae at 120 hpc (equivalent to 10 days post-hatching) could be the consequence of this physiological process at that time point of the developmental stage (27). However, the peak of cortisol observed at 120 hpc was not observed on the non-challenged group. On the other hand, the cortisol release could be the result of the starvation process, but both challenged and non-challenged groups were not fed along the experiment. Taken together, we hypothesize that the exposure to *V. anguillarum* is responsible for the high cortisol level obtained in our study at 120 hpc compared with the non-challenged group.

Importantly, the whole-body cortisol increase observed in our study was correlated with the high concentration of cortisone observed at 120 hpc. A previous report showed high concentration levels of cortisone after subjecting fish to stress (34), indicating that the surge of cortisol metabolites is probably a mechanism to downregulate cortisol into a non-active metabolite at physiological level (24). Therefore, a similar mechanism could take place in the sea bass larvae in our study at 120 hpc. In addition, no variations were registered on the cortisol precursors 17α-HP and 11-deoxycortisol. Thus, the absence of variations of direct cortisol precursors and the elevated levels of cortisol and cortisone raise the possibility that the machinery of cortisol synthesis would have been activated between 36 and 120 hpc. Thus, the high level of cortisol and cortisone (but not the cortisol precursors) observed in our study would be the result of the activation of cortisol negative feedback mechanisms. However, the non-increase of glucocorticoid secretion observed in our study at the first 36 hpc suggests that the presence of *V. anguillarum* was not recognized as an acute stressor at the first developmental stages. A previous report showed that sea bass larvae open their mouth from day 3 post-hatching onward and importantly, *V. anguillarum* can be tracked on the intestinal track from 2 h post-exposure onward (91). Therefore, the level of cortisol during the first 36 hpc indicates that sea bass larvae do not mount an immediate acute response even when *V. anguillarum* is in close contact with the host inner environment (91). This result agrees with ongoing research on the late inflammatory response to bacteria on axenic sea bass larvae (Galindo-Villegas and Bossier, unpublished results) and on the hypothalamic response of juvenile sea bream to *V. anguillarum* bacterin in which the response of peptides such as CRH binding protein and TRH did not take place until 48 h of *V. anguillarum* exposure, suggesting a delay in the stress perception at brain regulatory level (Khansari et al., unpublished results).

The receptors for cortisol are the glucocorticoid receptor (GR) and MR (26), although GR is considered the primary receptor for glucocorticoid action in teleosts (24). In our study, the increase of cortisol observed at 120 hpc did not vary the mRNA abundance of *gr1* nor *gr2*. Accordingly, a previous antecedent on sea bass larvae showed that the increase of cortisol was not associated with the augment of the expression of gr1 and gr2 (27). In mammals, these receptors have been found in almost all cells, indicating that almost all cells are targets for glucocorticoids (92). In teleosts, these receptors have been found in many tissues including immune cells (93, 94) and can explain the regulatory role of cortisol upon multiple aspects of immune defense including the secretion of pro- and anti-inflammatory cytokines. On the other hand, in our study, no variation was observed for *hsp70* throughout the time points tested. HSP70 plays a key role on the cortisol-GR ligand binding because its function as chaperone (95). A correlation between *gr* and *hsp70* mRNA abundance has been proposed, most probably associated with enhancement of cortisol sensitivity in immune cells (96). However, several studies have reported that the augment of cortisol does not affect the *gr* nor *hsp70* expression level (96, 97). Altogether, the lack of variation in the mRNA abundance of both *glucocorticoid receptors* and *hsp70* observed in our study suggests that the basal expression of these genes is physiologically adequate to mount an endocrine response in sea bass larvae challenged with *V. anguillarum*. The participation of other mechanisms promoted by cortisol remains to be elucidated.

Although there is no direct activation of an acute stress response during the first 36 hpc, an endocrine response could be indirectly activated through the immune system due to the bidirectional communication between neuroendocrine and immune systems. The neuro-immuno-endocrine network orchestrates the response to allostatic load in fish (24). Thus, it was reported in trout (*Oncorhynchus mykiss*) that cortisol treatment downregulates the expression of pro-inflammatory related genes in monocytes/macrophages primary cell culture stimulated with lipopolysaccharide (41). In sea bream, cortisol showed the ability to decrease the expression of pro-inflammatory cytokines both in leukocytes from the head kidney (42) and also in head kidney primary cell culture (HKPCC) (40). The same immunosuppresor cortisol effect was observed in sea bream HKPCC stimulated with inactivated *V. anguillarum* (35). This evidence, in combination with the results reported in our study, suggests that the high level of cortisol observed at 120 hpc in sea bass larvae challenged with *V. anguillarum* is a response mechanism attributed to the sensing of a biological threat related to the augmented pro-inflammatory response registered at 120 hpc. Glucocorticoids are considered to be mediators of the anti-inflammatory response by generating immunosuppression associated with nuclear factor-κB (NF-κB) by induction of the inhibitor of kappa B protein, trapping activated NF-κB in inactive cytoplasmic complexes (98). It is noticeable that pro-inflammatory genes such as *il-1β* and *il-8* (both modulated by *V. anguillarum*) have a NF-κB binding site (82). Therefore, the increase in markers associated with glucocorticoid synthesis could be related with the induction of an anti-inflammatory response in sea bass larvae challenged with *V. anguillarum*. The increase of glucocorticoids at 120 hpc is in accordance with the expression of innate immune response genes that are associated with a pro-inflammatory antibacterial response. This antecedent, together with the upregulation of genes associated with the anti-inflammatory response, suggests that in sea bass larvae infected with *V. anguillarum* a pro-inflammatory response at systemic level takes place, which then leads to the participation of other physiological mechanisms at systemic level to counteract the effect and the consequences of such response. This late systemic response could be the responsible for the high mortality observed in sea bass larvae challenged with *V. anguillarum*.

## Ethics Statement

All experiments were approved by the Ethical Committee of the Faculty of Veterinary Medicine and the Faculty of Bioscience Engineering, Ghent University (no. EC2015_02) and carried out in accordance with the recommendations of the European Union Ethical Guidelines for experimental animal care and other scientific purposes (2010/63/EU).

## Author Contributions

The conceptualization of the experiment was performed by FERL and PB. The methodology was originally proposed by FERL, JA, and PB. The experiments were carried out by FERL. The data analysis was performed by FERL, JA, EVV, and BA. FERL, KD, LT, and PB were the responsible for the acquisition of the financial support for the projects leading to this publication. FERL and LT wrote the original draft. All the authors corrected, read, and approved the final manuscript. The study was supervised by FERL, LT, and PB.

## Conflict of Interest Statement

The authors declare that the research was conducted in the absence of any commercial or financial relationships that could be construed as a potential conflict of interest.
